# Alpha-ketoglutarate ameliorates age-related osteoporosis via regulating histone methylations

**DOI:** 10.1038/s41467-020-19360-1

**Published:** 2020-11-05

**Authors:** Yuan Wang, Peng Deng, Yuting Liu, Yunshu Wu, Yaqian Chen, Yuchen Guo, Shiwen Zhang, Xiaofei Zheng, Liyan Zhou, Weiqing Liu, Qiwen Li, Weimin Lin, Xingying Qi, Guomin Ou, Cunyu Wang, Quan Yuan

**Affiliations:** 1 State Key Laboratory of Oral Diseases, National Clinical Research Center for Oral Diseases, West China Hospital of Stomatology, Sichuan University, Chengdu, China; 2 Laboratory of Molecular Signaling, Division of Oral Biology and Medicine, School of Dentistry and Jonsson Comprehensive Cancer Center, UCLA, Los Angeles, CA 90095 USA; 3 Present Address: Department of Stomatology, The First Affiliated Hospital of Zhengzhou University, Zhengzhou, China

**Keywords:** Methylation, Ageing, Bone, Ageing

## Abstract

Age-related osteoporosis is characterized by the deterioration in bone volume and strength, partly due to the dysfunction of bone marrow mesenchymal stromal/stem cells (MSCs) during aging. Alpha-ketoglutarate (αKG) is an essential intermediate in the tricarboxylic acid (TCA) cycle. Studies have revealed that αKG extends the lifespan of worms and maintains the pluripotency of embryonic stem cells (ESCs). Here, we show that the administration of αKG increases the bone mass of aged mice, attenuates age-related bone loss, and accelerates bone regeneration of aged rodents. αKG ameliorates the senescence-associated (SA) phenotypes of bone marrow MSCs derived from aged mice, as well as promoting their proliferation, colony formation, migration, and osteogenic potential. Mechanistically, αKG decreases the accumulations of H3K9me3 and H3K27me3, and subsequently upregulates BMP signaling and *Nanog* expression. Collectively, our findings illuminate the role of αKG in rejuvenating MSCs and ameliorating age-related osteoporosis, with a promising therapeutic potential in age-related diseases.

## Abstract (web summary)

α-ketoglutarate is an intermediate of the Krebs Cycle that was recently reported to extend lifespan in C.Elegans. Here, the authors show that administration of α-ketoglutarate to mice reduces age-related bone loss, by ameliorating senescence of bone-marrow derived mesenchymal stem cells.

## Introduction

Aging is characterized by the chronic and gradual deterioration of the functional capacities at the cellular, tissue, and organismal levels, making the individual more prone to various diseases, such as osteoporosis^1^. Osteoporosis is the systemic loss of bone mass and exhibits significant degradation in mechanical properties, which subsequently leads to an increased risk of bone fracture^2^. As the aging population is dramatically rising worldwide, the incidence of osteoporosis is significantly increasing as well, which creates a huge burden on public health^3^.

Recently, several therapies including caloric restriction, telomerase-based therapies, antioxidant supplementation, senolytic therapeutics, cellular reprogramming, and administration of epigenetic regulators (sirtuins activators, histone deacetylases inhibitors, noncoding miRNAs regulators, etc.) have been reported to have overall anti-aging effects, while the efficacy and biocompatibility of those therapies are still dubious^4^. As for the treatment of age-associated osteoporosis, FDA/Europe-approved treatments represented by bisphosphonates^5^ and recombinant human parathyroid hormone exert their therapeutic effects via either anabolic or anti-resorptive function^6^. However, their uses in long-term treatment are limited due to their adverse effects such as osteosarcoma, osteonecrosis of jaw, and atypical femur fractures^6,7^. Although there are various new drugs (Romosozumab^8^, Abaloparatide^9^, Cathepsin K inhibitors^10^, etc.) and novel therapeutic targets (Semaphorin 3A^11^, EphrinB2/EphB4^12^, etc.), lack of efficacy and occurrence of corresponding side effects impede their application in osteoporosis treatment^13^.

Alpha-ketoglutarate (αKG) is a crucial intermediate in the tricarboxylic acid (TCA) cycle, locating between succinyl-CoA and isocitrate. As a key point of anaplerotic reaction, αKG regulates ATP production and reduces equivalent (NAD+/NADH) generation in the TCA cycle, therefore influencing ROS level and immune system homeostasis^14^. Moreover, αKG is an important source of glutamine and glutamate, which are required for the synthesis of both amino acid and collagen^15^.

Administration of either αKG or its derivatives can promote bone development in the growing rats and lambs^16,17^, and protect bone loss induced by hormone deficiency^18–20^. Notably, recent studies reveal a pivotal role of αKG in maintaining the pluripotency of embryonic stem cells (ESCs)^21^ and in anti-aging therapy^22^. Administration of αKG extends the lifespan and delays the decline of rapid, coordinated body movement of worms, in a dose-dependent manner^22^. However, its influence on the age-related osteoporosis remains to be investigated.

In this study, we show that supplementation of αKG increases bone mass of aged mice, protects bone loss in adult mice, and accelerates bone regeneration of aged rodents. In vitro treatment with αKG ameliorates the age-related phenotypes of MSCs, along with promoting their proliferation, migration, and osteogenesis. Mechanistically, αKG decreases the accumulations of H3K9me3 and H3K27me3, and reduces their enrichments on the promoters of *Bmp2*, *Bmp4*, and *Nanog*.

## Results

### αKG increases the bone mass of aged mice

**Fig. 1:**
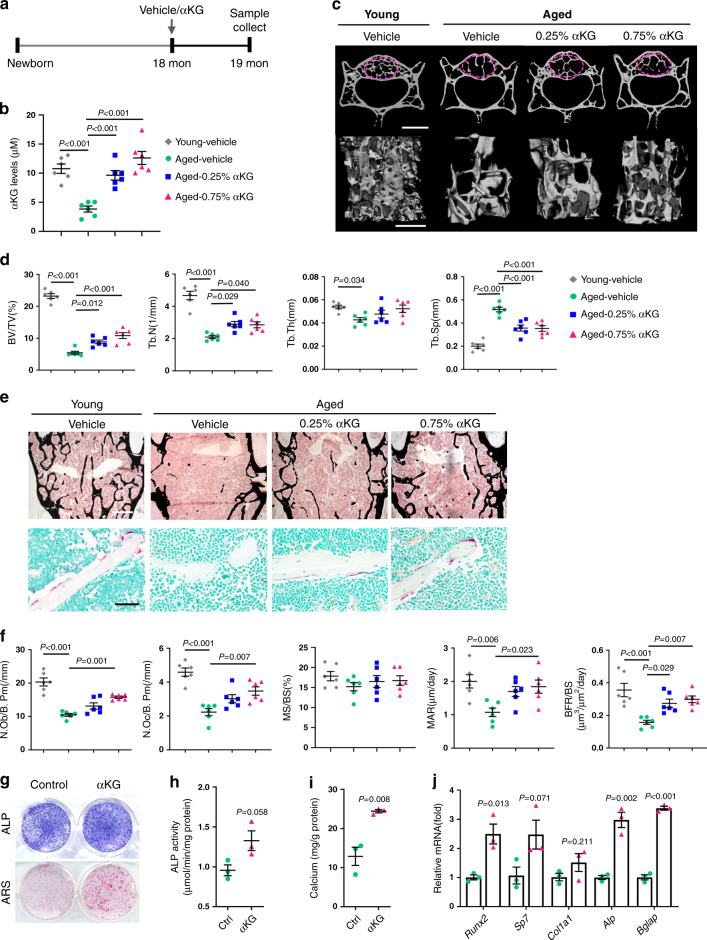
αKG increases the bone mass of aged female mice. **a** Schematic representation of αKG administration. **b** αKG administration increased circulating αKG levels of aged mice (*n* = 6, by one-way ANOVA with Tukey’s post hoc test). **c** Representative images of microCT reconstruction of lumber 4 (L4) vertebrae. Scale bar, 1 mm (upper) or 500 μm (lower). The magenta circles indicate the region of interest (ROI). **d** Quantitative microCT analyses of trabecular bone of L4 vertebrae (*n* = 6, by one-way ANOVA with Tukey’s post hoc test). Bone volume (BV/TV, %); trabecular number (Tb.N, 1/mm); trabecular thickness (Tb.Th, mm); trabecular separation (Tb.Sp, mm). **e** Representative Von Kossa staining and TRAP staining images of L4 vertebrae. Scale bar, 500 μm (upper) or 50 μm (lower). **f** Histomorphometric analyses of L4 vertebrae (*n* = 6, by one-way ANOVA with Tukey’s post hoc test). Number of osteoblasts (N.Ob/B.Pm, 1/mm); Number of osteoclasts (N.Oc/B.Pm, 1/mm); Mineral apposition rate (MAR, μm/day); Mineralizing surface per bone surface (MS/BS, %); Bone formation rate per bone surface (BFR/BS, μm^3^/μm^2^/day). **g** Representative images of alkaline phosphatase (ALP) and Alizarin Red S (ARS) staining of bone marrow MSCs isolated from the aged mice with or without αKG treatment. **h** Quantitative analysis of the ALP activity (*n* = 3, by two-tailed Student’s *t*-test). **i** Quantitative analysis of the mineralization (*n* = 3, by two-tailed Student’s *t*-test). **j** Quantitative RT-PCR results of mRNA expression of *Runx2, Sp7, Col1a1, Alp*, and *Bglap* in MSCs isolated from vehicle/αKG treated mice (*n* = 3, by two-tailed Student’s *t*-test). All data are shown as mean ± SEM.

To study the potential role of αKG in age-related osteoporosis, we first measured the circulating levels of αKG in aged rodents (mice, 18-months-old; rats, 24-months-old) and found they were significantly lower than those of young adults (3-months-old) (Supplementary Fig. 1a, b). Administration with 0.25 or 0.75% αKG in drinking water successfully restored the serum levels in aged mice (Fig. 1a, b), without a significant change of body weight (Supplementary Fig. 2a). MicroCT analyses showed that vertebral bone volume (BV/TV) of both female and male animals were elevated after αKG administration (Fig. 1c, d and Supplementary Fig. 3a, b). Consistently, the trabecular thickness (Tb.Th) and trabecular number (Tb.N) were increased, accompanied with decreased trabecular separation (Tb.Sp) (Fig. 1d and Supplementary Fig. 3b). Analogously, αKG administration also increased the bone mass of femur and tibia (Supplementary Fig. 4a–d). Analyses of femoral midshaft revealed an increased cortical bone mineral density (BMD) in αKG-treated mice, while cortical thickness (Ct.Th) was relatively even in all groups (Supplementary Fig. 4e, f).

Histomorphometric analyses confirmed the increased bone mass in αKG-treated groups, especially in 0.75% group (Fig. 1e and Supplementary Fig. 3c). αKG-treated mice displayed an increased number of osteoblasts (N.Ob/B.Pm) and osteoclasts (N.Oc/B.Pm) (Fig. 1f and Supplementary Fig. 3d), indicating a more active bone remodeling status. Higher mineral apposition rate (MAR) and bone formation rate (BFR) were also observed in αKG groups (Fig. 1f and Supplementary Fig. 3d).

Next, we isolated the MSCs from aged female mice treated with 1-month 0.75% αKG or vehicle, and cultured them in osteogenic induction medium. As determined by the alkaline phosphatase (ALP) and Alizarin Red S (ARS) staining, MSCs isolated from the αKG-treated mice exhibited a stronger osteogenic potential than that of the controls (Fig. 1g–i). In addition, the expression of the osteogenic-related genes, such as *Runx2*, *Sp7*, *Col1a1*, *Alp*, and *Bglap*, was upregulated in MSCs isolated from the αKG-treated mice (Fig. 1j).

### αKG attenuates age-related bone loss

**Fig. 2:**
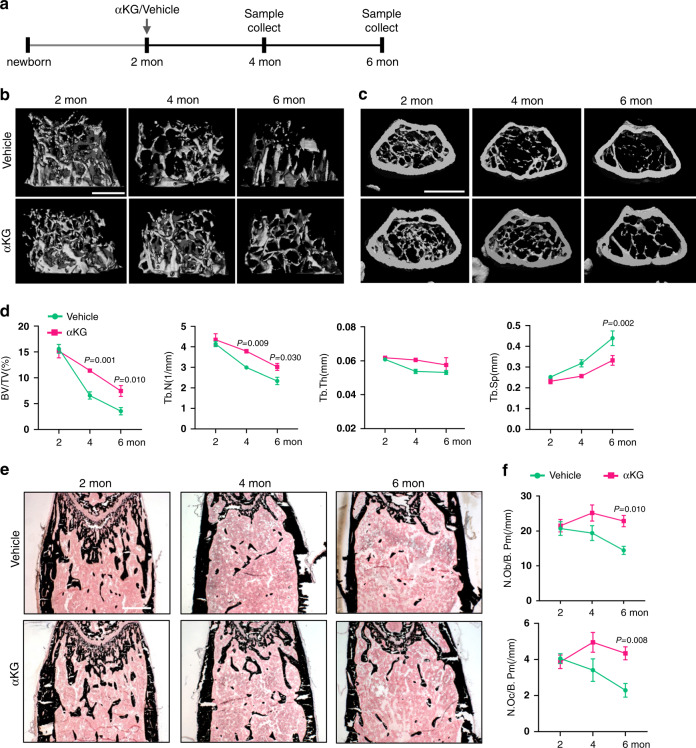
αKG attenuates age-related bone loss. **a** Schematic representation of αKG administration. **b** MicroCT reconstruction of distal ends of femurs in longitudinal direction. Scale bar, 500 μm. **c** MicroCT reconstruction of distal ends of femurs in horizontal direction. Scale bar, 1 mm. **d** Quantitative microCT analyses of distal femurs (*n* = 6). **e** Von Kossa staining of undecalcified sections of femurs. Scale bar, 500 μm. **f** Histomorphometric analyses of distal femurs (*n* = 6). All data are shown as mean ± SEM. The *P* values were calculated by two-way ANOVA with Sidak’s multiple comparisons test.

The trabecular bone mass of C57BL/6J mice reaches the peak when they become sexually mature (about 2-month-old) and declines sharply in the next few months^23,24^. We next sought to investigate if αKG is capable of attenuating age-related bone loss in adult female mice (Fig. 2a). No significant difference in body weight was observed during the experimental period (Supplementary Fig. 2b). Adult female mice suffered from a 77.5% reduction of femoral trabecular bone volume from 2-month-old to 6-month-old in the control group (Fig. 2b–d). Interestingly, supplementation of 0.25% αKG in the drinking water attenuated the trabecular bone loss. 75.3% and 49.1% BV/TV were maintained at 4-month-old and 6-month-old compared to 2-month-old mice, respectively (Fig. 2d). Consistently, the reduction in Tb.N and the progressive increase in Tb.Sp became moderate in the experimental group than the control group (Fig. 2d). In accordance with our findings in aged mice, the numbers of osteoblasts and osteoclasts were also increased in αKG-treated mice at 4-month and 6-month-old in comparison to the controls (Fig. 2e, f). Likewise, αKG decreased the age-related trabecular bone loss in tibiae and vertebrae (Supplementary Fig. 5a–f).

### αKG accelerates bone defect healing of aged rodents

**Fig. 3:**
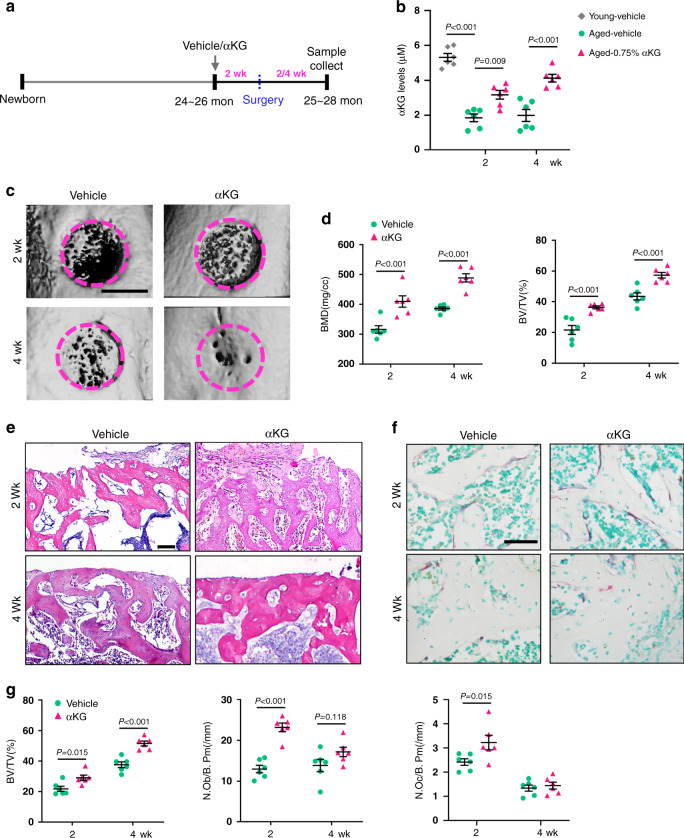
αKG accelerates the bone defect healing of aged rats. **a** Schematic representation of bone defect surgery and αKG administration. **b** Circulating αKG levels after αKG administration (*n* = 6, by one-way ANOVA with Tukey’s post hoc test). **c** Representative images of microCT reconstruction of femoral cortical bone defects at 2 and 4 weeks. The magenta dotted lines indicate the original position of the defect margin. Scale bar, 1 mm. **d** Quantitative microCT analyses of bone mineral density (BMD, mg/cc) and bone volume/total volume (BV/TV, %) within the original defect area (*n* = 6, by two-way ANOVA with Sidak’s multiple comparisons test). **e**, **f** Representative images of H&E staining and TRAP staining of femoral cortical bone defects. Scar bar, 100 μm (H&E), 50 μm (TRAP). **g** Histomorphometric analyses within the bone defect (*n* = 6, by two-way ANOVA with Sidak’s multiple comparisons test). Data are expressed as mean ± SEM.

The healing of bone defect is postponed along with aging^25^. Here we assessed the influence of αKG on bone healing of aged rats using a femoral defect model induced by drilling-hole injury. We started feeding aged rats with 0.75% αKG from 2 weeks prior to the surgery (Fig. 3a), and successfully elevated the circulating level of αKG (Fig. 3b). Both BMD and BV/TV were increased in the αKG-treated group compared with the controls at 2 weeks and 4 weeks after surgery (Fig. 3c, d). Histomorphometric analyses confirmed the increase of BV/TV, and numbers of osteoblasts and osteoclasts (Fig. 3e–g).

### αKG promotes proliferation and migration of aged MSCs

**Fig. 4:**
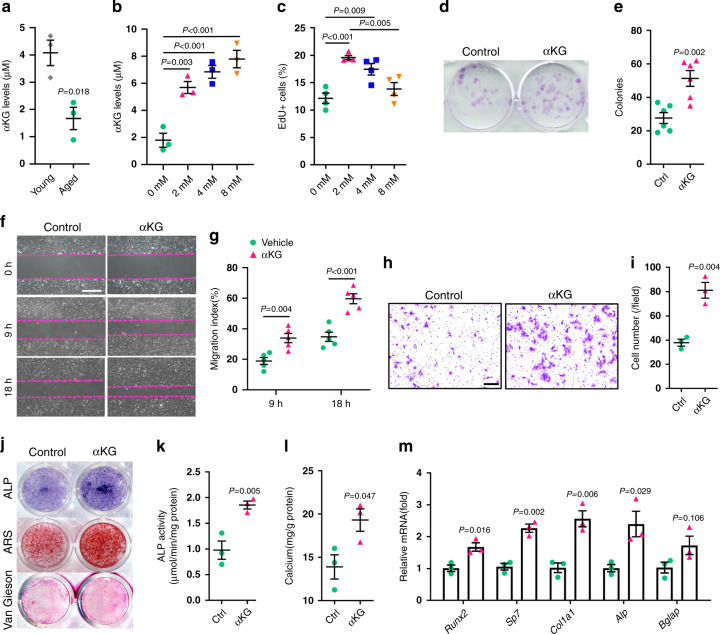
αKG promotes proliferation, migration, and osteogenesis of aged MSCs. **a** Intracellular αKG concentration in MSCs isolated from young (3-month-old) and aged (18-month-old) mice (*n* = 3, by two-tailed Student’s *t*-test). **b** Intracellular αKG concentration in aged MSCs in response to αKG treatment for 3 days (*n* = 3, by one-way ANOVA with Tukey’s post hoc test). **c** EdU incorporation in aged MSCs treated with different concentrations of αKG (*n* = 4, by one-way ANOVA with Tukey’s post hoc test). αKG treatment increased the number of EdU^+^ cells. **d** Crystal violet staining of colony-forming units of MSCs. **e** Quantitative analysis of colony-forming unit assay (colonies per well, *n* = 6, by two-tailed Student’s *t*-test). **f**, **g** Scratch assay of aged MSCs and quantitative analyses. Magenta dotted lines indicate start (0 h), half (9 h), and end (18 h) positions of MSCs after scraping (*n* = 5, by two-way ANOVA with Sidak’s multiple comparisons test). Scale bar, 500 μm. **h**, **i** Crystal violet staining of migrated MSCs from the upper chamber to bottom lower of the membrane and quantitative results (*n* = 3, by two-tailed Student’s *t*-test). Scale bar, 100 μm. **j** Representative images of ALP, ARS and Van Gieson staining of aged MSCs. **k** Quantitative analysis of the ALP activity in MSCs (*n* = 3, by two-tailed Student’s *t*-test). **l** Quantitative analysis of calcium mineralization in MSCs (*n* = 3, by two-tailed Student’s *t*-test). **m** Quantitative RT-PCR results of mRNA expression of *Runx2*, *Sp7*, *Col1a1*, *Alp,* and *Bglap* in MSCs treated with vehicle/αKG (*n* = 3, by two-tailed Student’s *t*-test). Data are expressed as mean ± SEM.

As the aging of MSC is accompanied with deterioration of proliferation capacity, colony-formation ability, migration activity, and osteogenic differentiation^26,27^, we sought to investigate whether αKG had any influence on those biological characteristics of aged MSCs. We observed a drop of intracellular αKG level in MSCs isolated from aged mice in comparison to those from young adults (Fig. 4a). Supplementation of αKG in culture medium successfully elevated the intracellular αKG levels (Fig. 4b) and increased EdU^+^ cells without affecting cell apoptosis (Fig. 4c and Supplementary Fig. 6a-c). In addition, αKG improved the colony formation capacity of aged MSCs (Fig. 4d, e). Next, we investigated the migration of MSCs through scratch assay and transwell assay in the presence or absence of 2 mM αKG. At 9 and 18 h after scratching, the αKG-treated group showed an accelerated speed of wound healing (Fig. 4f, g). Consistently, the migration activity of aged MSCs was also enhanced by αKG treatment in transwell assay (Fig. 4h, i).

### αKG stimulates osteogenic differentiation of aged MSCs

To evaluate the effect of αKG on osteogenic differentiation of aged MSCs in vitro, we isolated bone marrow MSCs from 18-month-old mice and cultured in osteogenic induction medium. αKG supplement augmented ALP activity after 7 days of osteogenic induction (Fig. 4j) and increased the formation of mineralized nodules after 3 weeks (Fig. 4j). These findings were confirmed by quantitative analyses of ALP activity and calcium deposition (Fig. 4k, l). Van Gieson staining also showed that αKG enhanced collagen production (Fig. 4j). Consistently, αKG treatment elevated the expression of the osteogenic-related genes, such as *Runx2*, *Sp7*, *Col1a1*, *Alp*, and *Bglap* (Fig. 4m).

### αKG ameliorates age-associated hallmarks of aged MSCs

**Fig. 5:**
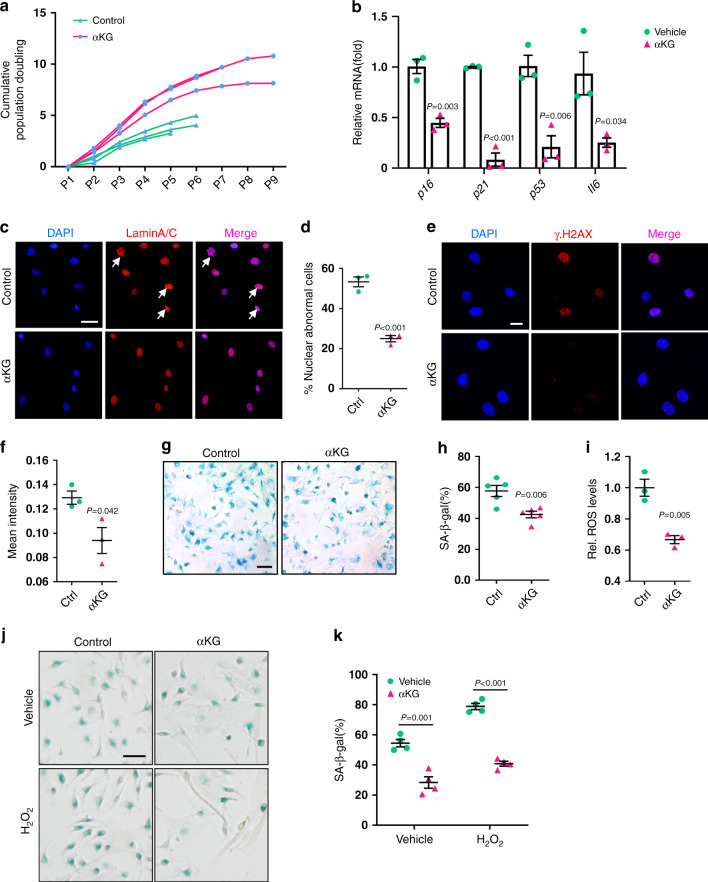
αKG ameliorates age-associated hallmarks of aged MSCs. **a** Cumulative population doubling (CPD) of aged MSCs (*n* = 3). **b** Quantitative RT-PCR results of senescence-associated genes (*p16, p21, p53, Il6*) in aged MSCs (*n* = 3). Cells were treated with 2 mM αKG for 3 days. **c**, **d** Immunofluorescence of Lamin A/C and quantification of nuclear abnormality in MSCs (*n* = 3). White arrows indicate blebbing in the nuclear envelope. Scale bar, 50 μm. **e**, **f** Immunofluorescence of γH2A.X and quantification of mean density in MSCs (*n* = 3). Scale bar, 20 μm. **g**, **h** Representative images of senescence-associated β-galactosidase (SA-β-gal) staining of aged MSCs and quantification of positive cells (*n* = 5). Scale bar, 50 μm. **i** ROS levels in aged MSCs (*n* = 3) were decreased after αKG supplement for 3 days. **j** SA-β-gal staining of aged MSCs stimulated with H_2_O_2_. Scale bar, 50 μm. **k** Quantification of SA-β-gal positive MSCs (*n* = 4). Data are expressed as mean ± SEM. The *P* values were calculated by two-tailed Student’s *t*-test, except **k**, which was analyzed by two-way ANOVA with Sidak’s multiple comparisons test.

To evaluate the effect of αKG on cell senescence, we isolated bone marrow MSCs from 18-month-old mice and treated with 2 mM αKG. The treated cells exhibited a longer lifespan and shorter population-doubling time, compared to the control cells (Fig. 5a). The expression of aging markers, such as *p16, p21, p53*, and *Il6*^27^, was significantly downregulated in response to αKG treatment (Fig. 5b). Besides, supplementation of αKG restored the nuclear envelope architecture (Fig. 5c, d), as confirmed by the immunofluorescence of progerin^28^ (aberrant prelamin A isoform). Phosphorylation of Ser-139 of histone H2A.X molecules (γ-H2A.X), a common indicator of DNA damage^29^, was also reduced by αKG treatment (Fig. 5e, f). In addition, as revealed by senescence-associated β-galactosidase (SA-β-gal) staining, the percentage of positive cells was decreased in the αKG group (Fig. 5g, h).

Next, we examined the ROS levels in MSCs 3 days after αKG treatment. The results showed a remarkable reduction in ROS levels in αKG-treated MSCs compared to the controls (Fig. 5i). We then applied H_2_O_2_ to exacerbate cell senescence and confirmed that αKG was capable of alleviating MSCs senescence induced by ROS (Fig. 5j, k).

### αKG downregulates the global H3K9me3 and H3K27me3 levels

**Fig. 6:**
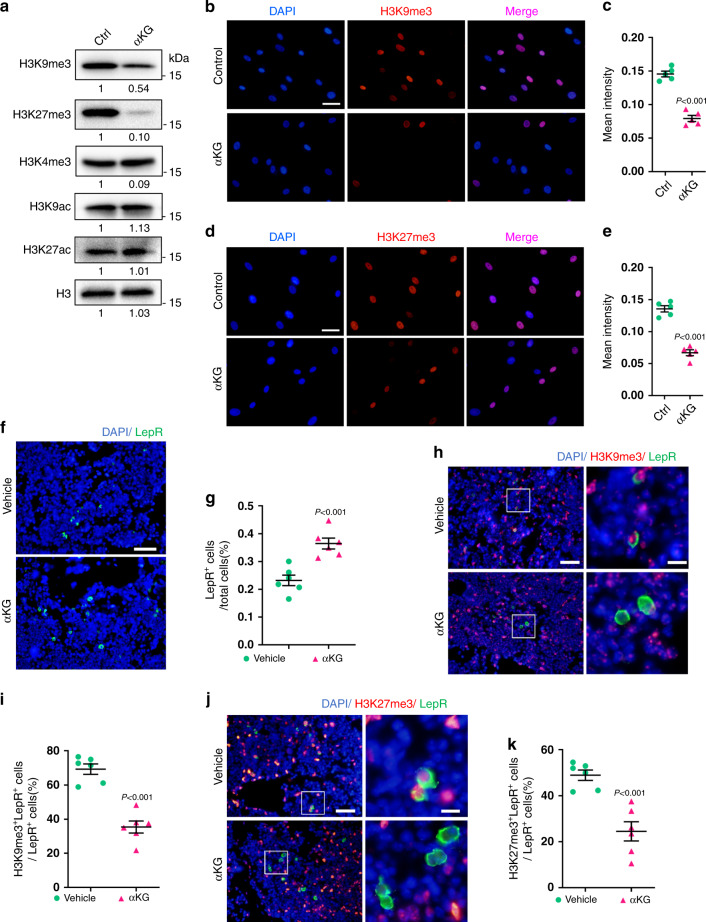
αKG decreases H3K9me3 and H3K27me3 abundance in aged MSCs. **a** Western blot analyses of H3K9me3, H3K27me3, H3K4me3, H3K9ac, and H3K27ac in aged MSCs treated with 2 mM αKG for 3 days. **b**, **c** Immunofluorescence staining of H3K9me3 and quantification (*n* = 5). Aged cells were treated with 2 mM αKG for 3 days. Scale bar, 50 μm. **d**, **e** Immunofluorescence staining of H3K27me3 and quantification (*n* = 5). Scale bar, 50 μm. **f**, **g** Immunofluorescence staining and quantification of LepR^+^ cells in vertebrae from aged mice fed with αKG for a month (*n* = 6). Scale bar, 50 μm. **h**, **i** Double staining and quantification of LepR-positive and H3K9me3-positive MSCs in vertebrae from aged mice fed with αKG for a month (*n* = 6). Scale bar, 50 μm (left) or 10 μm (right). **j**, **k** Double staining and quantification of LepR-positive and H3K27me3-positive MSCs in vertebrae from aged mice fed with αKG for a month (*n* = 6). Scale bar, 50 μm (left) or 10 μm (right). Data are presented as mean ± SEM. The *P* values were calculated by two-tailed Student’s *t*-test.

Given the role of histone modifications in the aging process^30–32^, we sought to investigate whether αKG exerted its function by modifying histone marks. Western blot analysis showed that αKG supplementation significantly reduced H3K9me3 and H3K27me3 levels in the aged MSCs isolated from 18-month-old mice, while levels of H3K4me3, H3K9ac, and H3K27ac remained unchanged (Fig. 6a). The downregulation of H3K9me3 and H3K27me3 was confirmed by immunofluorescence staining (Fig. 6b–e).

Next, we performed immunofluorescence staining of LepR, a bone marrow MSC marker^33,34^, on vertebral sections from aged mice. αKG administration increased LepR^+^ cell pools in vivo, indicating a relatively enlarged MSCs population in the bone marrow (Fig. 6f, g). Notably, the percentage of MSCs which were double labeled with LepR and H3K9me3 was significantly lower compared to the controls (Fig. 6h, i). The percentage of H3K27me3-stained LepR^+^ MSCs was also reduced by αKG administration (Fig. 6j, k).

### αKG regulates BMP pathway through histone methylations

**Fig. 7:**
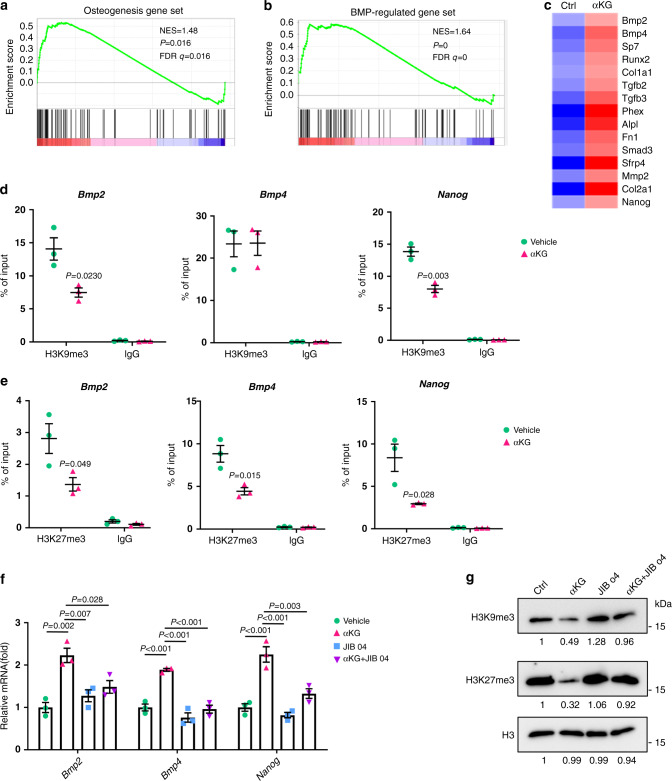
αKG regulates BMP pathway. **a**, **b** Gene set enrichment analysis (GSEA) showed increased expression of osteogenesis and BMP-regulated genes in αKG-treated MSCs. Cells isolated from aged mice were treated with 2 mM αKG for 48 h. **c** Heatmap of representative genes. **d** ChIP-qPCR assay revealed that αKG decreased the occupancy of H3K9me3 in the promoter regions of *Bmp2 and Nanog* (*n* = 3, by two-tailed Student’s *t*-test). **e** ChIP-qPCR showed that αKG decreased the occupancy of H3K27me3 in the promoter regions of *Bmp2, Bmp4,* and *Nanog* (*n* = 3, by two-tailed Student’s *t*-test). **f** Quantitative RT-PCR (*n* = 3, by one-way ANOVA with Tukey’s post hoc test). Histone demethylases inhibitor JIB 04 prohibited the effect of αKG on promoting *Bmp2, Bmp4,* and *Nanog* expression. **g** Western blot analyses of H3K9me3 and H3K27me3 in aged MSCs after supplementation of αKG and JIB 04. Data are shown as mean ± SEM.

Next, we performed RNA sequencing (RNA-seq) to examine transcriptome profiles in aged MSCs treated with or without αKG. Gene set enrichment analysis (GSEA) validated the upregulated expression of osteogenic marker genes (Fig. 7a). Of note, we find a significantly elevated expression of BMP-regulated genes, including *Bmp2* and *Bmp4* (Fig. 7b, c). We also found that the expression of *Nanog*, a master regulator of stem cell potency^35^, was increased (Fig. 7c).

Next, we examined the effect of αKG on accumulations of H3K9me3 and H3K27me3 at promoter regions of *Bmp2*, *Bmp4*, and *Nanog* by chromatin immunoprecipitation (ChIP) assay. The enrichment of H3K9me3 at the promoter regions of *Bmp2* and *Nanog* were reduced in the aged MSCs isolated from 18-month-old female mice when treated with αKG (Fig. 7d). Consistently, αKG treatment reduced H3K27me3 occupancy at the promoters of *Bmp2* and *Bmp4*, as well as in the promoter region of *Nanog* (Fig. 7e).

Notably, the mRNA levels of *Bmp2, Bmp4, Nanog* in aged MSCs were upregulated after αKG treatment, while this effect was largely abolished after the supplement of JIB 04 (Fig. 7f), a selective inhibitor of Jumonji histone demethylases^36^. Meanwhile, the mRNA expression levels of H3K9me3-modifying and H3K27me3-modifying enzymes remained unchanged after αKG treatment (Supplementary Fig. 7a, b). Besides, the application of 1 μM JIB 04 for 3 days counteracted the downregulation effects of αKG on H3K9me3 and H3K27me3 at protein level via western blot analyses (Fig. 7g). Those findings support that the effect of αKG on MSCs aging relies, at least partially, on the regulation of histone modifications.

## Discussion

As the aging population growing worldwide, discovering therapies to rejuvenate the deteriorated physiological functions and ameliorate the age-associated symptoms has become an intriguing issue in scientific fields^4,37^. αKG is a crucial intermediate of the Kreb Cycle that is recently reported to have potential anti-aging effects. The data from in vitro studies support that αKG plays roles in maintaining the pluripotency of ESCs^21^, and accelerating the primordial differentiation of primed human pluripotent stem cells and germ cells^38,39^. Furthermore, Huang et al. revealed that αKG treatment successfully extended the lifespan of elegans^22^. Specifically, Lin et al. reported that maintenance of αKG levels via caloric restriction played a crucial role in preserving brain physiology in aged rats^40^. Another in vivo study showed that dietary supplementation of αKG stabilized redox state and improved blood vessel elasticity in aged mice^41^. Moreover, Cynober et al. noted that, ornithine-ketoglutarate improved appetite, increased body weight gain, and accelerated wound healing, thus improving the clinical outcome of chronic malnutrition in elderly patients^42,43^.

To our knowledge, this is the first study that investigates the effect of αKG on skeleton aging using a model of age-related osteoporosis. A previous study reported that, αKG level in human serum experienced a notable decline upon aging^44^. Likewise, circulatory αKG concentration was found to be reduced in middle‐aged mice (10‐month‐old) compared with young mice (2‐month‐old)^45^. Consistently, we observed such reduction in aged rodents compared to younger organisms. αKG supplementation successfully compensates for this decline, in which case provides the rationality for us to explore the potential therapy of skeleton aging through αKG administration.

In this study, we illustrated that supplementation of αKG in drinking water not only increased the bone mass of physiologically aged mice, but also attenuated the rapid bone loss in adult mice. We showed that the female C57BL/6J mice exhibited a 77% reduction of femoral trabecular bone volume from 2-month old to 6-month old, which is close to the data of Glatt et al.^23^, but higher than some other reports. Previous studies show cancellous bone loss begins in early adulthood in C57BL/6 mice (2–3-month-old), earlier than that of BMD^23,24,46^. Notably, different mouse strains exhibit different patterns in bone loss^47–49^. C57BL/6J mice experience bone loss as early as 2 months of age, while BALB/c mice wouldn’t show significant reduction until 7 months^50^. Female mice also undergo a sharper decline in BV/TV. C57BL/6J female mice may experience a reduction of about 70% femoral trabecular bone from 2-month old to 6-month old^23^, while male mice lose 25–35% of the value^23,24^. In addition, the methods of Micro CT scanning and analysis may affect the data. In this study, we used a Scanco μCT50 scanners. For long bone, trabecular bone was analyzed starting from 160 μm away from the growth plate and extending 1200 μm proximally. As for vertebra, the region 160 μm below the cranial and above the caudal growth plate in the vertebral body was selected for analysis.

In the past few decades, scientific perspectives on the pathogenesis of osteoporosis have undergone a transformation from estrogen-centric to aging-centric^51^. The intrinsic cause of osteoporosis is considered to be the age-related changes in multiple organs and tissues (immune system, skeleton system, and endocrine system, etc.), particularly the dysregulation of MSC lineage commitment in the aged bone environment^52^. In this study, we proved the protective effects of αKG in MSCs aging. Of note, deteriorated capacity of proliferation, migration, and osteogenesis of MSCs towards aging is well-characterized by previous studies^26,27^. Here we found that, αKG could largely maintain the proliferation vitality, enhanced the migration of MSCs in vitro, and promoted osteogenic differentiation of aged MSCs. Moreover, with the treatment of αKG in vitro, the lifespan of aged MSCs was extended and the senescence-associated hallmarks of MSCs were significantly downregulated, including the mRNA level of SA genes (*p16, p21, p53,* and *Il6*), markers of nuclear architecture (Lamin A/C) and DNA damage marker γ-H2A.X, and SA-β-gal activities. As aging increases the susceptibility of MSCs towards ROS^53^, our data reveal a restrained ROS level and cell senescence upon αKG treatment, either in vehicle condition or under the exacerbated ROS stress induced by H_2_O_2_. These results are consistent with previous studies in different cell types and organisms^54^.

αKG has been reported to bind and block the mitochondrial ATP synthase, leading to the inhibition of mechanistic target of rapamycin (mTOR) signaling, through which αKG extended worm lifespan^22,55^. It is believed that, whether αKG favors ESCs and pluripotent stem cells (PSCs) towards self-renewal or differentiation largely relies on the pluripotent stage, which is affected by oxidative phosphorylation (OXPHOS) level, induction circumstance, etc^38,56,57^. However, TeSlaa et al. showed that αKG did not promote differentiation of primed PSCs via inhibiting ATP synthase. Instead, the role of αKG in epigenetic regulation was suggested to be responsible for favoring the naive state of PSCs or ESCs, or accelerating induced primed PSC differentiation^21,38^.

Epigenetic alterations are regarded as hallmarks of cellular senescence or organismal aging^58^. And histone modifications are documented as an essential component^59,60^. Our data show αKG relieves the overall burden of H3K9me3 and H3K27me3, with little effects on levels of H3K4me3, H3K9ac, and H3K27ac. H3K9me3 and H3K27me3 are two critical histone modifications that are closely associated with cell senescence and organismal aging, and age-associated osteoporosis as well^61–63^. Previous in vitro studies showed that a relatively low methylation level of H3K9me3 and H3K27me3 is a linchpin to maintain the pluripotency of ESCs^21^. Ye et al. have reported that in the bone marrow of aged mice, the overall accumulations of H3K9me3^+^ and H3K27me3^+^ cells are significantly elevated compared with the young controls in vivo^32^. Here, both our in vitro and in vivo data show that the number of H3K9me3^+^ and H3K27me3^+^ MSCs were reduced after αKG administration.

αKG is believed to serve as a co-substrate for Fe(II)/αKG-dependent dioxygenases, which are widely present in all living organisms^64,65^. These enzymes play important roles in various biological processes including the post-translational modification of collagen, fatty acid metabolism, oxygen sensing, DNA and RNA repair, demethylations related to epigenetic regulation^66^. Of note, the JMJC family of histone demethylases can be classified into αKG-dependent dioxygenases, using αKG as an indispensable cofactor^67,68^. As histone methylation status is manipulated by the counteraction of histone methyltransferases and demethylases, the JMJC family of histone demethylases has been reported as the crucial regulator of MSC differentiation^69^. Here, we demonstrate downregulation of H3K9me3 and H3K27me3, without affecting the expression of histone-modifying enzymes, which implies a more active status of histone demethylases upon αKG administration.

In summary, we demonstrate that αKG protects age-related osteoporosis and rejuvenates aged MSCs. It reduces overall H3K9me3 and H3K27me3 levels, and their occupancy at promoters of *Bmp2*, *Bmp4,* and *Nanog*, highlighting the therapeutic potential of αKG in age-related osteoporosis.

## Methods

### Animals

C57BL/6J mice and Sprague Dawley rats were purchased from the Chengdu Dossy Experimental Animals CO. LTD. Animals were randomly allocated into either control or αKG group and housed in specific pathogen-free facilities under a 12-h light and 12-h dark cycle. Temperature (23 ± 2 °C) and humidity (55%) were held constant in animal housing. All animals were allowed free access to food and clean water in the absence or presence of αKG (K1128, Sigma). Both the vehicle water and αKG solution were adjusted to 7.3 by the addition of NaOH^16,17^. Approvals for all the protocols were obtained from the Subcommittee on Research and Animal Care (SRAC) of Sichuan University.

### μCT analysis

Bone tissues (femur, tibia, and vertebra) were dissected and fixed in 10% paraformaldehyde for 48 h and stored in 70% ethanol at 4 °C before being processed. The microCT imaging system (μCT50, SCANCO Medical) was applied to evaluate trabecular bone and bone regeneration. The specimens were well placed in a cylindrical holder (14 mm in diameter) with the long axis of the bone tissues perpendicular to the X-ray beam and a spatial resolution of 8 μm (55 kV, 114 mA, 500 ms integration time). Thereafter, volumetric reconstructions and analyses were performed by built-in software. For trabecular bone analysis, the region of interest (ROI) was selected according to the anatomical structure of those bone tissues. One hundred and fifty slices were chosen as ROIs for long bone (distal femur and tibia), starting from 20 slices beneath the growth plates. As for vertebrae (L4), around 300–400 slices of the vertebral body were selected as the ROIs for analysis (160 μm below the cranial and above the caudal growth plate regions). Bone volume fraction (BV/TV), trabecular number (Tb.N), trabecular separation (Tb.Sp), and trabecular thickness (Tb.Th) were calculated within the ROIs. For bone regeneration analysis, ROI was defined as a cylindrical area situating at the initial bone defect. Bone volume fraction (BV/TV) and the bone mineral density (BMD) within the delimited ROIs were evaluated.

### Histomorphometric analyses

Processing of undecalcified bone specimens and cancellous bone histomorphometry were performed as described^25,70^. Bone specimens (femur, tibia, and vertebra) were dehydrated and embedded in methylmethacrylate. Sections (5 μm in thickness) were prepared using a microtome (#RM2235, Leica) microtome and were stained by the von Kossa/nuclear fast red method. For hematoxylin and eosin (HE) and tartrate-resistant acid phosphatase (TRAP) staining, bone specimens were decalcified in 10% EDTA for 2 weeks at room temperature. After serial dehydration in a series of ethanol (70–100%), samples were embedded in paraffin. Sections (5 μm in thickness) were processed by a microtome (#RM2235, Leica) ready for staining. Histomorphometric measurements were performed using OsteoMeasure software (OsteoMetrics, Decatur, GA)^71^.

### Cell culture

Primary MSCs were isolated from aged C57BL/6J mice at 18 months old by flushing the bone marrow of tibiae and femurs according to the published protocol^72^. Cells were cultured in minimum essential medium α (α-MEM) supplemented with 10% heat-inactivated fetal bovine serum (Gibco) and 1% penicillin/streptomycin (HyClone), with or without supplementation of cell-permeable αKG (K1128, Sigma). MSCs were grown on 100 cm^2^ dish (Corning) at 37 °C in an atmosphere of 5% CO_2_. MSCs were passaged at 90% confluence^73^.

### Osteogenic induction and alkaline phosphatase activity assay

In vitro osteogenesis of MSCs was induced using the osteogenic differentiation medium, containing 50 μg/ml ascorbic acid, 5 mM β-glycerophosphate, 100 nM dexamethasone (all from Sigma)^25^, supplemented with or without 2 mM αKG.

On day 10, cells were fixed with 4% polyoxymethylene for 30 min. After washing cells with PBS for three times, cells were incubated at 37 °C with 0.1 M Tris buffer (pH 9.3) consisting 0.25% naphthol AS-BI phosphate (#N2125, Sigma) and 0.75% Fast Blue BB (D9805, Sigma). Images of stained plates were obtained using a scanner (#V330, EPSON). Quantification of alkaline phosphatase (ALP) activity was performed using a commercial kit, according to the instructions (#P0321, Beyotime). And the optical density was examined via spectrophotometer (Thermo Fisher Scientific) at 405 nm.

### Mineralization assay and Van Gieson staining

Alizarin Red S (ARS) staining was performed as described^71^. Briefly, cells were fixed after 21-day induction of osteogenic differentiation with 4% polyoxymethylene for 30 min. After washing cells with PBS for three times, cells were stained with 1% Alizarin red S (pH 4.2, Sigma-Aldrich) for 20 min. Images of stained plates were obtained using a scanner (V330, EPSON). For mineralization quantification, mineralized matrix stained with alizarin red were destained with 10% cetylpyridinium chloride for 30 min continuous gentle shaking. The calcium concentration was evaluated by detecting the optical density at 562 nm (Thermo Fisher Scientific), and converting those data using the standard calcium curve in the same solution.

Van Gieson Staining was performed using a commercial kit (G1338, Solarbio), according to manufacturer’s instruction.

### Bone defect surgery

The rodent bone defect model was established through the surgery on the long bone of aged rats. In brief, aged female rats at 24-month-old were anesthetized through intraperitoneal injection of a mixture of ketamine (100 mg kg^−1^) and xylazine (10 mg kg^−1^). Besides, buprenorphine (0.05 mg kg^−1^) was administrated before operation to minimize suffering and pain. The anterior-distal surfaces of the femurs were exposed by blunt dissection of the quadriceps after skin incision. A 1.4 mm defect was created with a round bur (Komet, Germany) operating at 10,000 r.p.m. under irrigation of saline, and then ample saline was used to rinse off bone fragments. Muscles were stitched using interrupted 5-0 Monocryl sutures and skin closures were completed subsequently with 3-0 sutures. Bone tissues were collected 2 or 4 weeks after surgery. Upon specimen harvest, femoral samples were fixed in 4% paraformaldehyde for 48 h at 4 °C and were transferred into 70% ethanol and stored at 4 °C for further experiments.

### αKG level evaluation and reactive oxygen species (ROS) assay

Circulating αKG levels in serum and intracellular αKG levels were measured by using an αKG assay kit (ab83431, Abcam) according to the manufacturer’s instructions. After 3 days of treatment with αKG, ROS levels in cells were measured using cellular ROS detection kit (ab186027, Abcam) following the manufacturer’s instructions.

### Proliferation assay and apoptosis assay

MSCs were treated with different concentrations of αKG (0/2/4/8 mM) for 3 days, and then were plated on 8-well culture slides overnight to allow for attachment. For 5-ethynyl-2′-deoxyuridine (EdU) incorporation assay, cells were cultured in the presence of 10 μM EdU and different concentrations of αKG (0/2/4/8 mM) for 24 h, and then fixed and permeabilized for imaging according to the manufacturer’s instructions (C10337, Invitrogen).

Apoptotic cells were detected using FITC Annexin V Apoptosis Detection Kit I according to the manufacturer’s instructions (#556547, BD Biosciences).

### Colony formation assay

For colony formation assay, MSCs were seeded at 200 cells per well in 6-well plates with or without 2 mM αKG. Medium was changed every 3 days, and cells were fixed and stained with 0.5% crystal violet in 98% ethanol on day 10. Colonies containing more than 50 cells were counted in each well.

### Migration assay

In vitro migration activity of MSCs was evaluated via scratch assay and transwell assay. For the scratch assay, MSCs were cultured in 6-well plates until reaching confluence and began to be starved 24 h before scratching. Cells were scraped rapidly across the center of the plates via a pipette tip (20–200 μl). Photographs were taken under an inverse phase-contrast microscope at 0/9/18 h after cell scratch, and the average width of the scratched gap was measured in triplicate wells by the ImageJ software (v.1.6.0). For transwell assay, the transwell inserts with 8 μm membrane (#3422, Corning) were plated in the wells of 24-well plates. Six hundred microliter of 10% FBS medium with or without αKG was added to the lower chamber, and MSCs (5 × 10^4^ cells/well) were seeded into the upper chamber in a serum-free medium. After 12 h incubation at 37 °C, medium in the lower and upper chamber was discarded, and the cells lining in the upper side of the membrane were removed by cotton swabs. MSCs migrated to the lower surface of the membrane were fixed with 4% paraformaldehyde for 30 min and stained with 0.05% crystal violet for 1 h. Randomly selected and photographed five fields for each sample at ×200 magnification under a microscope. The number of stained cells per field was counted and calculated.

### Lifespan analysis

MSCs obtained from aged mice (18–20 months old) were seeded (1 × 10^4^ cells/cm^2^) in 100 mm dishes. The adherent cells were counted after 3–4 days to quantify the initial number. At 90% confluence, the cells were digested in trypsin (HyClone, GE), counted and reseeded at 5 × 10^3^ cells/cm^2^ in 6-well plates, treated with vehicle/αKG. This procedure was repeated until MSCs reached their maximal life span, indicated by the growth arrest when the cells unable to become confluent within 4 weeks of culturing. Cumulative Population Doubling (CPD) was according to the formula PD = log2(Nh/Ni), CPD = sum, *I* = 1….nPD, where Nh = harvested number of cells, Ni = initial number of cells, and *n* = number of passage.

### Senescence-associated-*β*-galactosidase assay

The SA-β-gal activity was detected using the in situ β-galactosidase staining kit (#RG0039, Beyotime) following the manufacturer’s instructions. MSCs isolated from aged mice (18–20 months old) were seeded into 6-well culture plates and treated with or without αKG for 3 days. The cells were washed twice with PBS and fixed with the 4% paraformaldehyde for 15 min. After incubation with the SA-β-gal detection solution at 37 °C for 2 h, the cells were washed and analyzed under the microscope (Leica) on a scale of 400× for ten random sites. To evaluate the effect of αKG on H_2_O_2_-induced senescence, cells were treated with 100 μM H_2_O_2_ for 2 h and maintained in 10 μM H_2_O_2_ for an additional 48 h in the absence or presence of αKG.

### Immunofluorescence staining

Cells were grown on glass slides in 24-well plates. Upon harvest, cells were fixed with 4% paraformaldehyde and at room temperature (RT) for 20 min. Then, cells were permeabilized with 0.1% Triton X-100 in PBS at 4 °C for 10 min. Cells were incubated with 4% BSA in PBS for 30 min at 37 °C to block nonspecific interaction. Primary antibodies were added into the wells and cells were incubated at 4 °C overnight. Primary antibodies used in this study were as follows: polyclonal anti-γH2A.X (#9718S, CST, 1:200), polyclonal anti-Lamin A/C (#4777S, CST, 1:200), polyclonal anti-H3K9me3 (#ab8898, Abcam, 1:500), polyclonal anti-H3K27me3 (#9733S, CST, 1:1000). Next day, primary antibodies were removed, and cells were rinsed with PBS for 5 min. Then we incubated cells at room temperature for 1 h with the corresponding secondary antibody and washed with PBS for three times afterward. Cells were mounted using an Antifade Mounting Medium with DAPI (#H-1200, VECTOR).

For immunofluorescence double staining, freshly dissected bone tissues were fixed in 4% paraformaldehyde for 2 days and decalcified in 10% EDTA for 2 weeks. Slides were incubated at 99 °C for 20 min for antigen retrieval using sodium citrate buffer. Then, sections were incubated with the combination of polyclonal goat anti-LepR (#AF497, R&D, 1:200) and polyclonal anti-H3K9me3 (#ab8898, Abcam, 1:500), polyclonal anti-H3K27me3 (#9733S, CST, 1:1000) at 4 °C overnight. After washing with PBS solution for three times, the slides were incubated with secondary antibodies of donkey anti-goat FITC (#bs-0294D-FITC, Bioss, 1:500) and donkey anti-rabbit Alexa Fluor 555 (#A0453, Beyotime, 1:500) in dark for 1 h at room temperature. Images of these slides were acquired with a microscope (IX81, Olympus), and quantitative analyses were conducted via ImageJ software.

### Quantitative RT-PCR

Total RNA was extracted from cultured MSCs using Trizol (Invitrogen) according to the manufacturer’s instruction, and NanoDrop 2000 (Thermo Fisher Scientific) was used to quantify the purity and concentration of RNA. One microgram cDNA of each sample was obtained using the PrimeScript RT reagent Kit with gDNA Eraser (Takara). The real-time PCR was performed using SYBR Premix Ex Taq II (Takara) in CFX96 Real-Time System (Bio-Rad) following the manufacturer’s instruction, and the relative expression of target gene was normalized by housekeeping gene (*Gapdh)* using a 2^−ΔΔCt^ method subsequently. The primers used for qPCR are presented in Supplementary Table 1.

### Western blot

Proteins were isolated in RIPA buffer (Pierce, Rockford, IL) on ice. Lysates were separated by electrophoresis on 12% SDS-PAGE polyacrylamide gels. Then, samples were electro-transferred to PVDF membranes (0.22 μm, Millipore) using a wet transfer method and blocked with 5% BSA for 1 h at room temperature. Then, membranes were incubated overnight at 4 °C with primary antibodies of H3 (#4499, CST, 1:2000), H3K9me3 (#ab8898, Abcam, 1:1000) and H3K27me3 (#9733S, CST, 1:1000), H3K4me3 (#9751, CST, 1:1000), H3K9ac (#9649, CST, 1:1000), H3K27ac (#8173, CST, 1:1000), respectively. The next day, blots were incubated with horseradish peroxidase (HRP)-conjugated secondary antibodies (Jackson Immuno Research, West Grove, PA) at room temperature for 1 h. Finally, ECL reagent (Millipore) was used for the visualization and detection of antibody-antigen complexes. The uncropped gel images are shown in Supplementary Fig. 8.

### RNA sequencing and GSEA

Total RNAs from aged MSCs with or without αKG treatment were extracted by Trizol reagent and purified using poly-T oligo-attached magnetic beads (Cat. 61006, Thermo). We used NEBNext® Ultra™ RNA Library Prep Kit for Illumina® (NEB, USA) following the manufacturer’s recommendations to generate sequencing libraries^74^. Samples were then subjected to Illumina HiSeq 3000. FastQC (v0.11.5) and FASTX toolkit (0.0.13) were used to control the quality of RNA-seq data, which were then mapped to Mus musculus reference genomes (NCBI build 37, Jul 2007, mm9) using HISAT2 (v.2.0.4). Ballgown software (v.3.4.0) was performed to identify differentially expressed genes and transcripts. Genes were considered significantly differentially expressed if showing ≥1.5 fold change and *P* value < 0.05.

For GSEA, the gene sets for osteogenesis and BMP pathway were obtained from Qiagen or GSEA online database, respectively. We imported our gene list of interest into the GSEA software (http://www.broad.mit.edu/GSEA, v.4.0.2) and examined whether this given gene set showed statistically significant. *P* values were computed using a bootstrap distribution created by resampling gene sets of the same cardinality.

### ChIP assay

The enzymatic shearing of chromatin from MSCs was performed using the EZ-Zyme™ Chromatin Prep Kit (#17-375, Millipore) according to the manufacturer’s instructions. Briefly, cells were incubated in 1% formaldehyde for 10 min at room temperature to crosslink chromatin, and then stopped by the glycine. Then, the samples were collected and enzymatic sheared step by step with the mixed buffers supplied in this kit. Chromatin was fragmented to 200–400 bp, as confirmed by the results of agarose gel electrophoresis. Subsequently, enzymatic sheared chromatins were CHIP-ed using the EZ-Magna ChIP™ HiSens Chromatin Immunoprecipitation Kit (#10-10461, Millipore) according to the manufacturer’s protocol. The antibodies for ChIP assay were H3K9me3 (#ab8898, Abcam, 4 μg/test), H3K27me3 (#9733S, CST, 4 μg/test) and IgG (supplied in the kit, 4 μg/test). Precipitated DNA samples were quantified with real-time PCR and data are presented as the percentage of input DNA.

### Statistics and reproducibility

All values were expressed as mean ± SEM. Statistically significant differences were assessed by unpaired two-tailed Student’s *t*-test for comparison between two groups, one-way ANOVA followed by Tukey’s post hoc test and two-way ANOVA followed by Sidak’s multiple comparisons test for multiple comparisons. A *P* value < 0.05 was considered statistically significant. Results are from three independent experiments.

### Reporting summary

Further information on research design is available in the Nature Research Reporting Summary linked to this article.

## Supplementary information

Supplementary Information

Reporting Summary

## Data Availability

The RNA-seq datasets have been submitted to the NCBI database under the accession number GSE139496. Other databases used in the study are Mus musculus reference genomes (NCBI build 37, Jul 2007, mm9), Qiagen (https://www.qiagen.com) and GSEA online database (https://www.gsea-msigdb.org/gsea/index.jsp). The authors declare that all other data supporting the findings of this study are available within the article and its Supplementary information files. Source data are provided with this paper.

## Source data

Source Data
