# Supplementary material for: Alpha-ketoglutarate ameliorates age-related osteoporosis via regulating histone methylations
Source: Nat Commun. 2020 Nov 5;11:5596. doi: 10.1038/s41467-020-19360-1 (PMC7645772; doi:10.1038/s41467-020-19360-1)
Supplement: Supplementary file 1 — Supplementary Information [file 41467_2020_19360_MOESM1_ESM.pdf]

Alpha-Ketoglutarate Ameliorates Age-related Osteoporosis via Regulating Histone Methylations

Wang et al.

Table of contents

Supplementary Figure 1 Page 2

Supplementary Figure 2 Page 2

Supplementary Figure 3 Page 3

Supplementary Figure 4 Page 4

Supplementary Figure 5 Page 5

Supplementary Figure 6 Page 6

Supplementary Figure 7 Page 7

Supplementary Figure 8 Page 8

Supplementary Table 1 Page 9

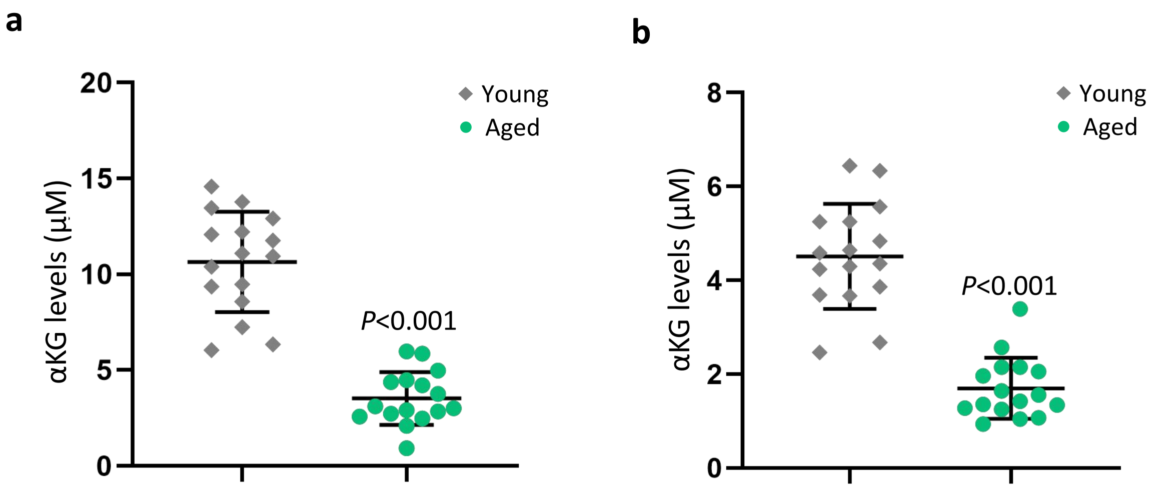

Supplementary Figure 1. Circulating level of αKG declines upon aging. (**a**) Serum αKG levels in young (3-mon-old) and aged (18-mon-old) mice (n=16). (**b**) Serum αKG levels in young (3-mon-old) and aged (24-mon-old) rats (n=16). Results are shown as mean ± SEM. The *P* values were calculated by two-tailed Student's *t* test.

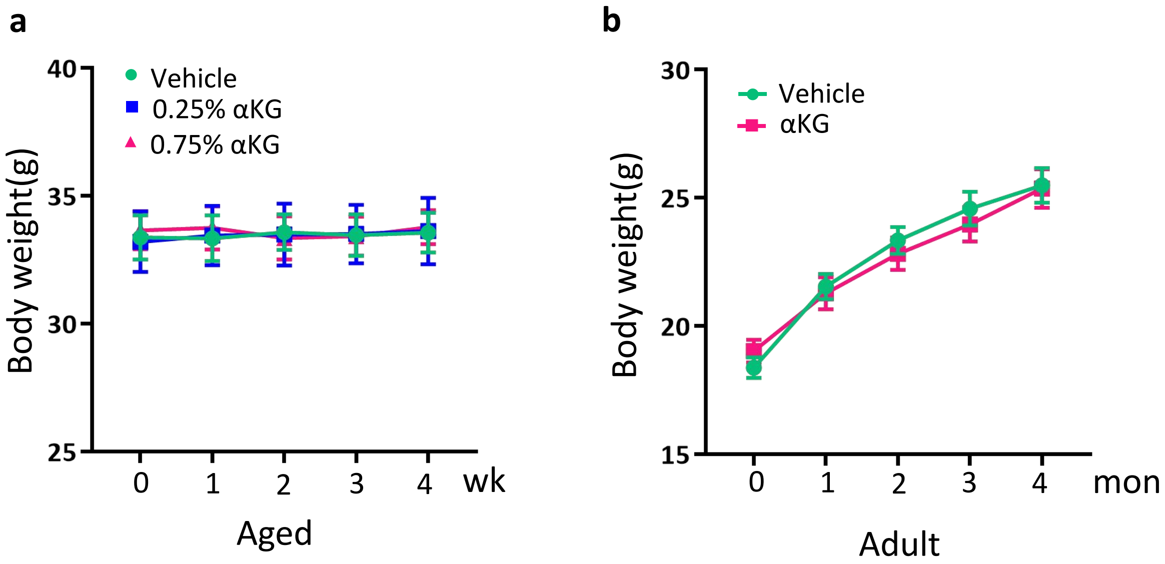

Supplementary Figure 2. Body weight during αKG administration. (a) Body weight of aged mice during 1-month αKG supplementation (n=6). **(b)** Body weight of adult mice with αKG supplementation starting from 2-month-old (n=6). Results are shown as mean ± SEM.

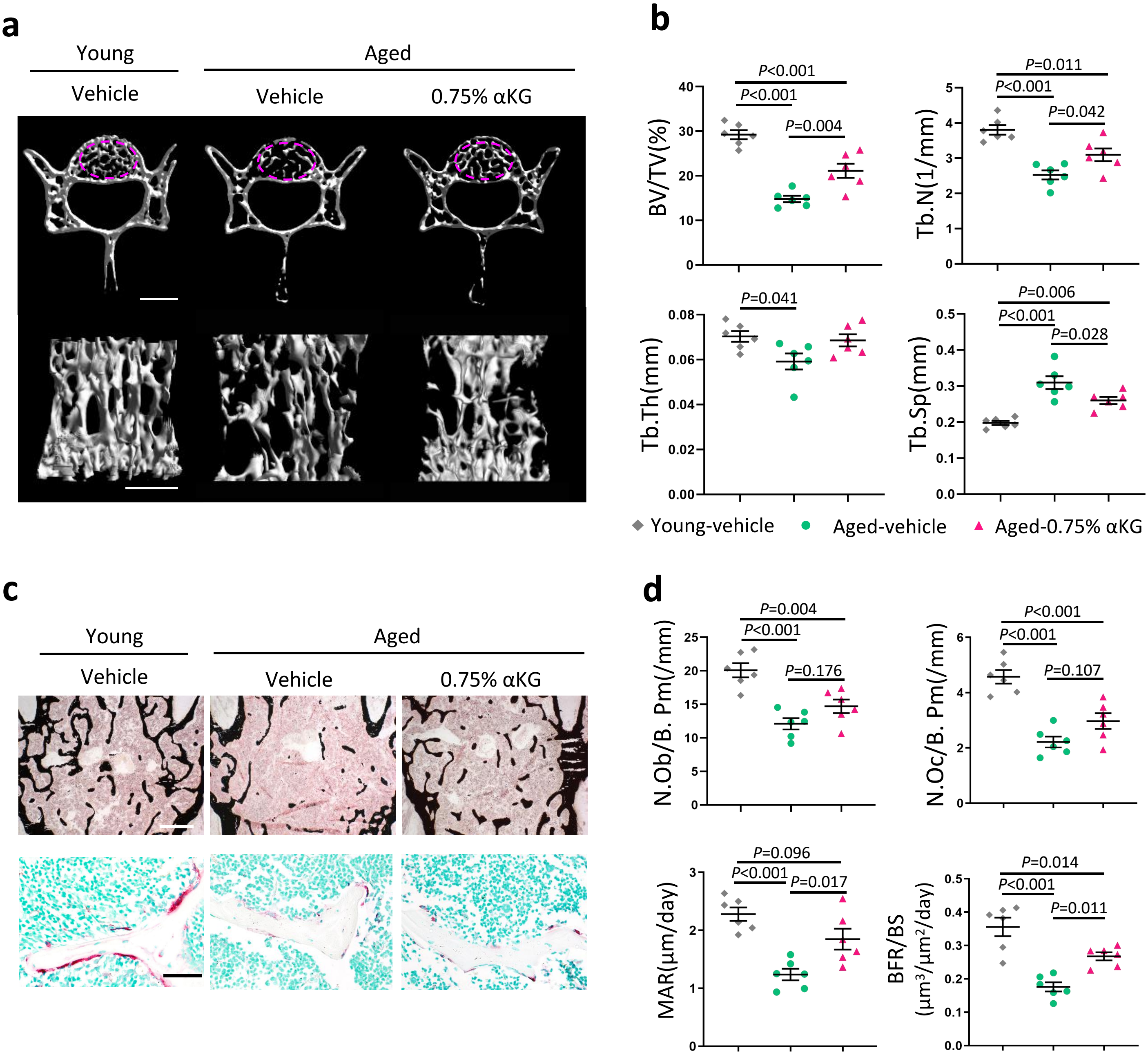

Supplementary Figure 3. αKG increases the bone mass of aged male mice. (**a**) Representative images of microCT reconstruction of L4 vertebrae. Scale bar, 1mm (upper) or 500μm (lower). The pink circles indicate the region of interest (ROI). (**b**) Quantitative microCT analyses of trabecular bone of L4 vertebrae (n=6). (**c**) Von Kossa staining and TRAP staining images of L4 vertebrae. Scale bar, 500μm (upper) or 50μm (lower). (**d**) Histomorphometric analyses of vertebrae (n =6). Data are shown as mean ± SEM. The *P* values were calculated by one-way ANOVA with Tukey's *post hoc* test.

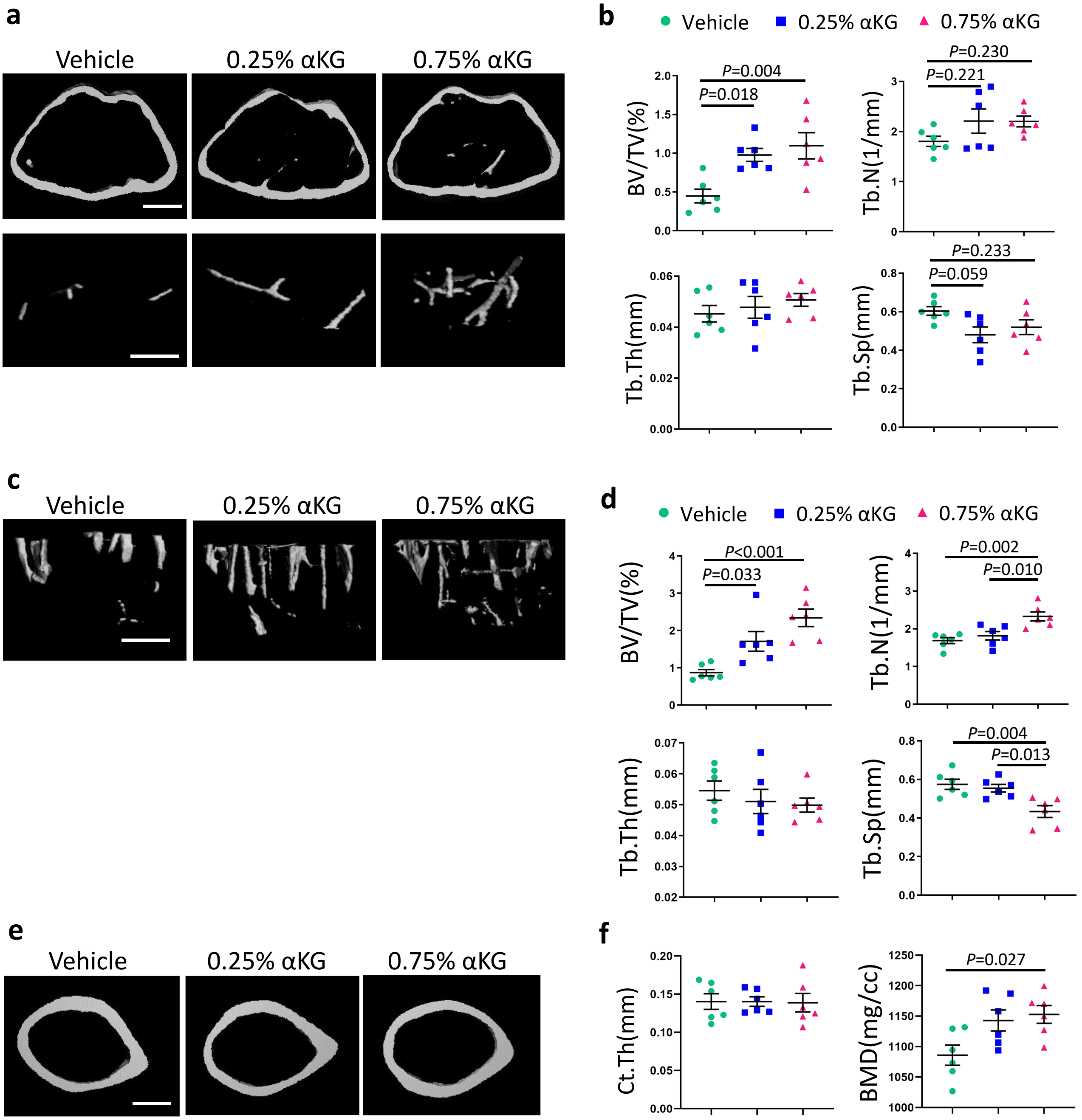

Supplementary Figure 4. αKG increases the bone mass of femurs and tibiae in aged female mice. (**a**) Representative images of microCT reconstruction of femurs. Scale bar (both), 500µm. (**b**) Quantitative microCT analyses of trabecular bone of distal femurs (n = 6). (**c**) Representative images of microCT reconstruction of mouse tibiae. Scale bar, 500µm. (**d**) Quantitative microCT analyses of trabecular bone in tibiae (n = 6). (**e**) Representative microCT images of midshaft cortical bone of femurs. Scale bar, 500µm. (**f**) Quantitative microCT analyses of cortical bone of femurs (n = 6). Data are shown as mean ± SEM. The *P* values were calculated by one-way ANOVA with Tukey's *post hoc* test.

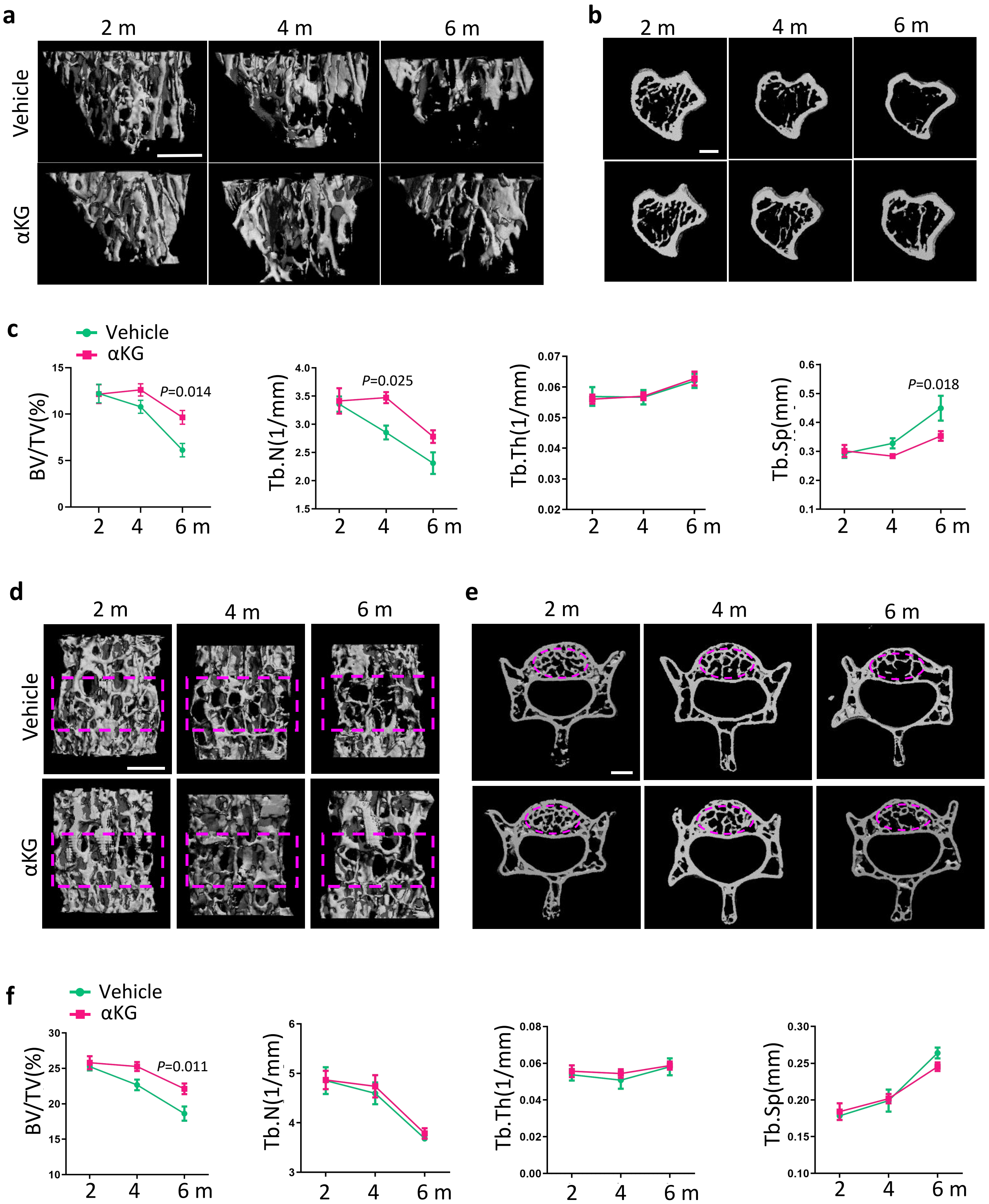

Supplementary Figure 5. αKG prevents age-related bone loss of tibiae and vertebrae. (a, b) Representative images of microCT reconstruction of proximal tibiae in longitudinal and horizontal direction. Scale bar, 500μm. (**c**) Quantitative microCT analyses of trabecular bone in tibiae (n = 6). (**d**) Representative images of microCT reconstruction of L4 vertebrae in longitudinal direction. Scale bar, 500μm. (**e**) Representative images of microCT reconstruction of L4 vertebrae in horizontal direction. Scale bar, 1mm. (**f**) Quantitative microCT measurements of L4 vertebrae. Data are shown as mean ± SEM. The *P* values were calculated by two-way ANOVA with Sidak's *multiple comparisons* test.

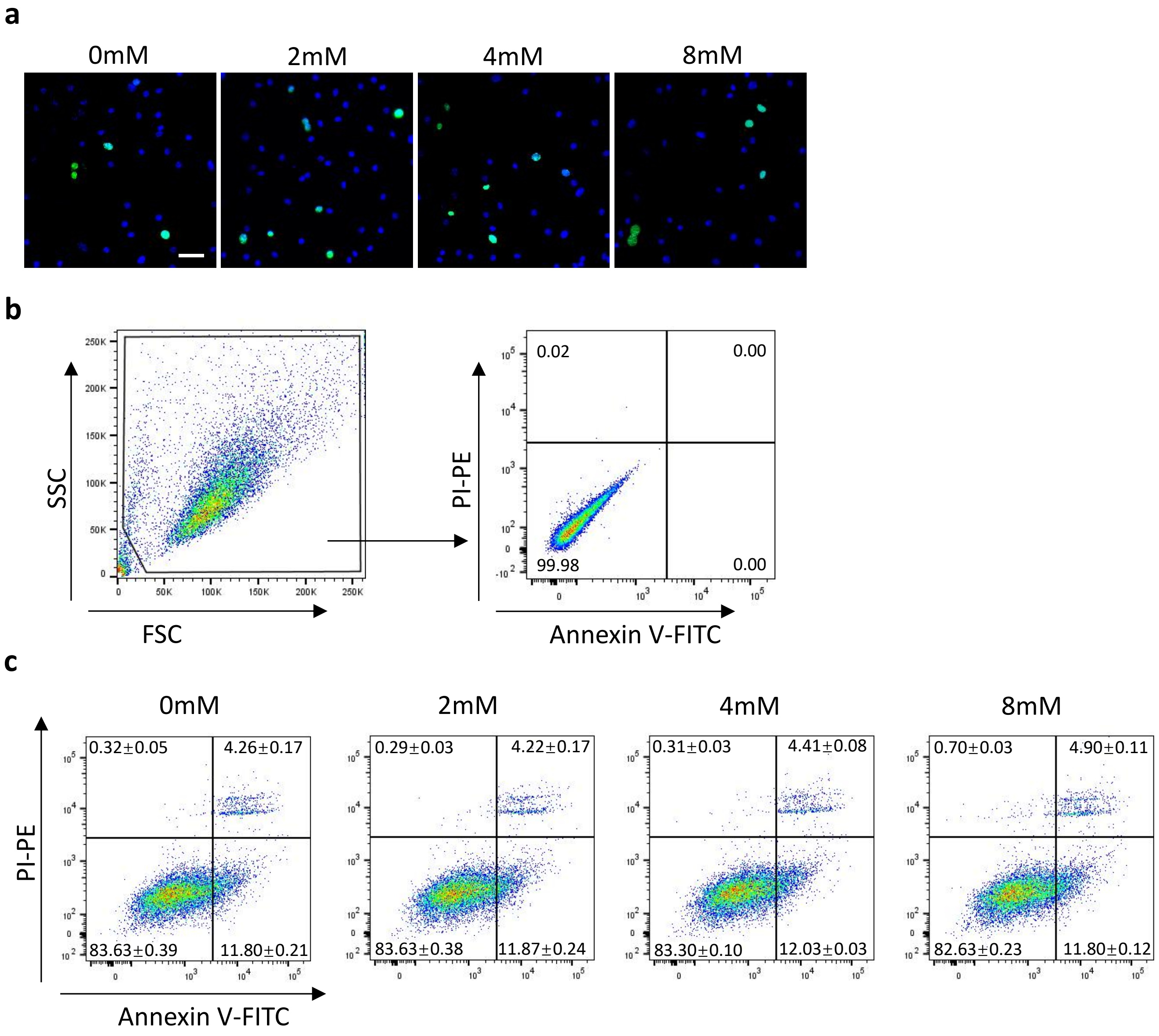

Supplementary Figure 6. αKG promotes cell proliferation without affecting apoptosis. (**a**) Immunofluorescence staining of EdU⁺ MSCs after αKG (0/2/4/8 mM) treatment for 3 days. Scale bar, 50 μm. (**b**) Gating strategy for apoptotic MSCs. FSC, forward scatter; SSC, side scatter. (**c**) FACS assay of apoptotic cells after αKG (0/2/4/8 mM) treatment for 3 days. Data are shown as mean ± SEM (n=3).

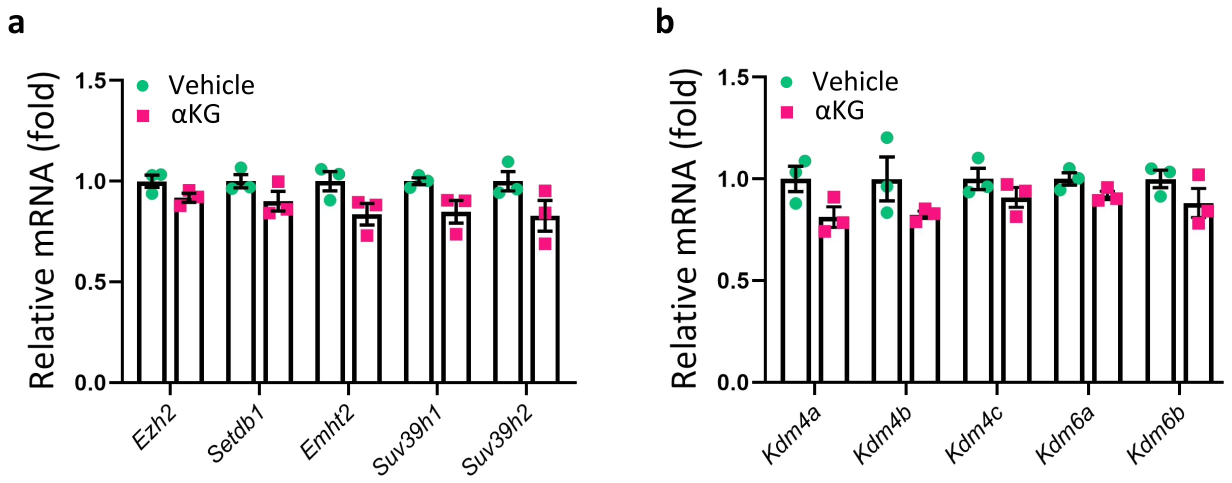

Supplementary Figure 7. Expression of histone-modifying enzymes. (**a**) Quantitative RT-PCR results of histone methylases *Ezh2*, *Setdb1*, *Emht2*, *Suv39h1* and *Suv39h2* (n=3). Aged MSCs were treated with 2mM αKG for 3 days. (**b**) Quantitative RT-PCR results of histone demethylases *Kdm4a*, *Kdm4b*, *Kdm4c*, *Kdm6a* and *Kdm6b* (n=3). Results are shown as mean ± SEM, no difference by Student's *t* test.

Figure 6a

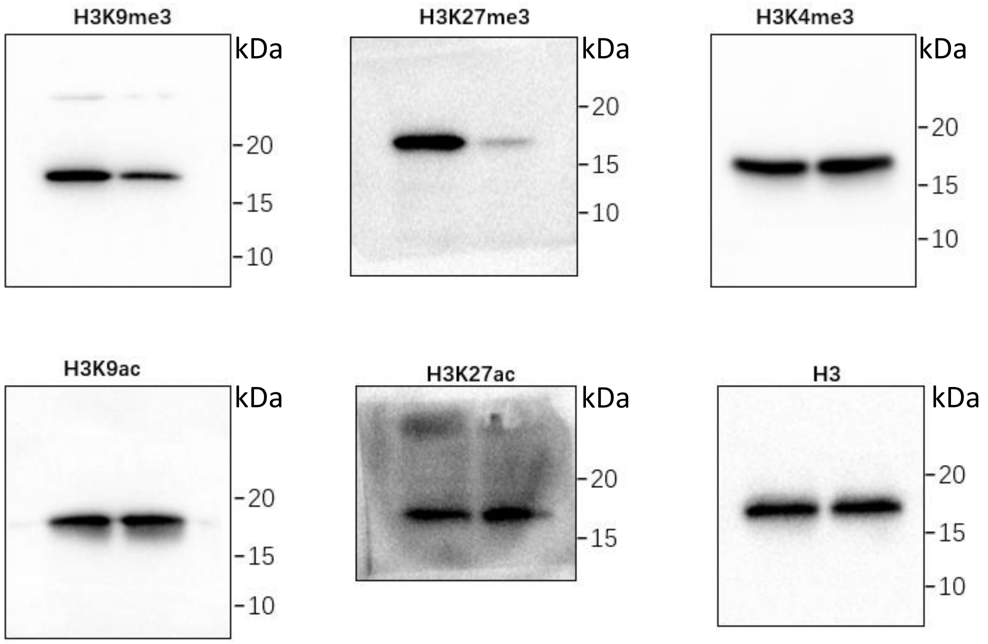

Figure 7g

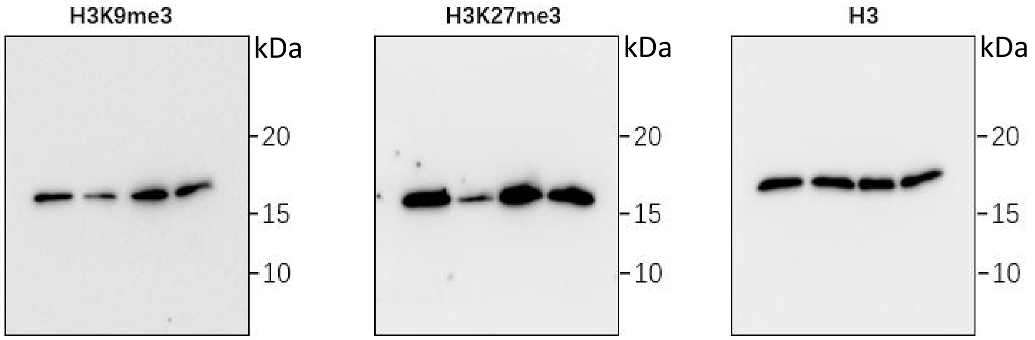

Supplementary Figure 8. Uncropped images of western blot.

Supplementary Table 1. Primer Sheet

Gene	Forward (5’-3’)	Reverse (5’-3’)
Gapdh	ACTGAGGACCAGGTTGTC	TGCTGTAGCCGTATTCATTG
Il6	TAGTCCTTCCTACCCCAATTTCC	TTGGTCCTTAGCCACTCCTTC
p16	CGTGAACATGTTGTTGAGGC	GCAGAAGAGCTGCTACGTGA
p53	CTCTCCCCCGCAAAAGAAAAA	CGGAACATCTCGAAGCGTTTA
p21	CCTGGTGATGTCCGACCTG	CCATGAGCGCATCGCAATC
Sp7	ATGGCGTCCTCTCTGCTTG	TGAAAGGTCAGCGTATGGCTT
Bglap	GAACAGACAAGTCCCACACAGC	TCAGCAGAGTGAGCAGAAAGAT
Col1a1	GGCCATTGTGTATGCAGC	ACATGTTCAGCTTTGTGGACC
Runx2	GGTACTTCGTCAGCATCCTATCAG	GCTTCCGTCAGCGTCAACAC
Alp	AACCCAGACACAAGCATTC	GCCTTTGAGGTTTTTGGTCA
Bmp2	AACACCGTGCGCAGCTTCCATC	CGGAAGATCTGGAGTTCTGCAG
Bmp4	GCCGAGCCAACACTGTGAGGA	GATGCTGCTGAGGTTGAAGAGG
Nanog	AGGACAGGTTTCAGAAGCAGA	CCATTGCTAGTCTTCAACCACTG
Ezh2	AGCACAAGTCATCCCGTTAAAG	AATTCTGTTGTAAGGGCGACC
Setdb1	GCCTACAGCAAGGAACGCATTC	GTTAGCTGGTGGCAGGCACATT
Ehmt2	GGAGCCAACATCAATGCCGTAG	TAGACAGGTGGAGCCATCCTCT
Suv39h1	CTTTGCCACAAGAACCATCTGGG	GCCAAAGTTGGAGTCCATTCGG
Suv39h2	CCATAAACGCTGGAGAAGAGCTG	CTGCAAGTCTCGGCTCCACATT
Kdm4a	TGCGGCAAGTTGAGGACAGTCT	GGATTCACAGAAAGGTCCAGTGC
Kdm4b	TGTCTCACCCATCTATGGAG	ATGTCTAGGATGGTCCTCAG
Kdm4c	TGTGAAGCAGCAGGTAGCGAGT	GTCTGCCAAAGGTGGATGAGAG
Kdm6a	AGCACAGAGGAGCCGTGGAAAA	GTCGTTCACCATTAGGACCTGC
Kdm6b	AGTGAGGAAGCCGTATGCTG	AGCCCCATAGTTCCGTTTGTG
