# Supplementary material for: Alpha-ketoglutarate ameliorates age-related osteoporosis via regulating histone methylations
Source: Nat Commun. 2020 Nov 5;11:5596. doi: 10.1038/s41467-020-19360-1 (PMC7645772; doi:10.1038/s41467-020-19360-1)
Supplement: Supplementary file 2 — Reporting Summary [file 41467_2020_19360_MOESM2_ESM.pdf]

nature research

Corresponding author(s): Quan Yuan

Last updated by author(s): Sep 10, 2020

Reporting Summary

Nature Research wishes to improve the reproducibility of the work that we publish. This form provides structure for consistency and transparency in reporting. For further information on Nature Research policies, see our Editorial Policies and the Editorial Policy Checklist.

Statistics

For all statistical analyses, confirm that the following items are present in the figure legend, table legend, main text, or Methods section.

n/a	Confirmed	
[ ]	[x]	The exact sample size (n) for each experimental group/condition, given as a discrete number and unit of measurement
[ ]	[x]	A statement on whether measurements were taken from distinct samples or whether the same sample was measured repeatedly
[ ]	[x]	The statistical test(s) used AND whether they are one- or two-sided<br>*Only common tests should be described solely by name; describe more complex techniques in the Methods section.*
[ ]	[x]	A description of all covariates tested
[ ]	[x]	A description of any assumptions or corrections, such as tests of normality and adjustment for multiple comparisons
[ ]	[x]	A full description of the statistical parameters including central tendency (e.g. means) or other basic estimates (e.g. regression coefficient) AND variation (e.g. standard deviation) or associated estimates of uncertainty (e.g. confidence intervals)
[ ]	[x]	For null hypothesis testing, the test statistic (e.g. F, t, r) with confidence intervals, effect sizes, degrees of freedom and P value noted<br>*Give P values as exact values whenever suitable.*
[x]	[ ]	For Bayesian analysis, information on the choice of priors and Markov chain Monte Carlo settings
[x]	[ ]	For hierarchical and complex designs, identification of the appropriate level for tests and full reporting of outcomes
[ ]	[x]	Estimates of effect sizes (e.g. Cohen's d, Pearson's r), indicating how they were calculated

Our web collection on statistics for biologists contains articles on many of the points above.

Software and code

Policy information about availability of computer code

Data collection	OsteoMeasure(Ver 3.1.0.2),HISAT2 (v.2.0.4), imagej (v.1.6.0), Ballgown software (v.3.4.0), GSEA(v.4.0.2), BD FACSDiva(v8.0.3), Flowjo (Version 10.5.3)
Data analysis	Software: GraphPad Prism; version 8.00.

For manuscripts utilizing custom algorithms or software that are central to the research but not yet described in published literature, software must be made available to editors and reviewers. We strongly encourage code deposition in a community repository (e.g. GitHub). See the Nature Research guidelines for submitting code & software for further information.

Data

Policy information about availability of data

All manuscripts must include a data availability statement. This statement should provide the following information, where applicable:

- Accession codes, unique identifiers, or web links for publicly available datasets
- A list of figures that have associated raw data
- A description of any restrictions on data availability

The RNA-seq datasets have been submitted to the NCBI database under the accession number GSE139496. Other databases used in the study are Mus musculus reference genomes (NCBI build 37, Jul 2007, mm9), Qiagen (https://www.qiagen.com) and GSEA online database (https://www.gsea-msigdb.org/gsea/index.jsp). The authors declare that all other data supporting the findings of this study are available within the article and its Supplementary information files. The source data are provided.

Field-specific reporting

Please select the one below that is the best fit for your research. If you are not sure, read the appropriate sections before making your selection.

- [x] Life sciences
- [ ] Behavioural & social sciences
- [ ] Ecological, evolutionary & environmental sciences

For a reference copy of the document with all sections, see nature.com/documents/nr-reporting-summary-flat.pdf

Life sciences study design

All studies must disclose on these points even when the disclosure is negative.

Sample size	No statistical method was used to predetermine the sample size. Sample size was determined according to previously published paper in relevant field and prior experience in our laboratory(Weiqing Liu et al,Nature Communications, 2016; Yunshu Wu et al, Nature Communications, 2018).
Data exclusions	All inclusion/ exclusion criteria were pre-established and no samples or animals were excluded from the analysis.
Replication	All experiments were performed as technical or biological replicates as appropriate for the experimental design.
Randomization	Animals were randomly allocated into either control or experimental group.
Blinding	As treatment was administered in the drinking water, investigators were not able to carry out experiment with blinding during the administration period. Blinding was performed later in the outcome assessment by other investigators.

Reporting for specific materials, systems and methods

We require information from authors about some types of materials, experimental systems and methods used in many studies. Here, indicate whether each material, system or method listed is relevant to your study. If you are not sure if a list item applies to your research, read the appropriate section before selecting a response.

Materials & experimental systems

n/a	Involved in the study	
[ ]	[x]	Antibodies
[ ]	[x]	Eukaryotic cell lines
[x]	[ ]	Palaeontology and archaeology
[ ]	[x]	Animals and other organisms
[x]	[ ]	Human research participants
[x]	[ ]	Clinical data
[x]	[ ]	Dual use research of concern

Methods

n/a	Involved in the study	
[x]	[ ]	ChIP-seq
[ ]	[x]	Flow cytometry
[x]	[ ]	MRI-based neuroimaging

Antibodies

Antibodies used	anti-Υ H.AX (#9718S, CST, 1:200); anti-Lamin A/C (#4777S, CST, 1:200); anti-H3K9me3 (#ab8898, Abcam, 1:500); anti-H3K27me3 (#9733S, CST, 1:1000); H3K4me3 (#9751, CST, 1:1000); H3K9ac (#9649, CST, 1:1000); H3K27ac (#8173, CST, 1:1000);goat anti-LepR-biotin (#AF497, R&D, 1:200); anti-H3 (#4499, CST, 1:2000); donkey anti-goat FITC (#bs-0294D-FITC, Bioss, 1:500) and donkey anti-rabbit Alexa Fluor 555 (#A0453, Beyotime, 1:500) .
Validation	anti-Υ H.AX (#9718S, CST): Application:WB,IHC,IF,F; Species Reactivity: Human, Mouse, Rat, Monkey<br>anti-Lamin A/C (#4777S, CST): Application:WB,IP,IHC,IF,F; Species Reactivity: Human, Mouse, Rat, Monkey<br>anti-H3K9me3 (#ab8898, Abcam): Application:WB, IHC-P, ICC/IF, ChIP; Species Reactivity: Mouse, Rat, Human, Saccharomyces cerevisiae, Indian muntjac<br>anti-H3K27me3 (#9733S, CST): Application:WB,IHC,IF,F,ChIP; Species Reactivity: Human, Mouse, Rat, Monkey<br>H3K4me3 (#9751, CST): Application:WB,IHC,IF,F,ChIP; Species Reactivity: Human, Mouse, Rat, Monkey, D. melanogaster, S. cerevisiae<br>H3K9ac (#9649, CST): Application:WB,IP,IHC,IF,F,ChIP; Species Reactivity: Human, Mouse, Rat, Monkey, Zebrafish<br>H3K27ac (#8173, CST): Application:WB,IF,F,ChIP; Species Reactivity: Human, Mouse, Rat, Monkey<br>goat anti-LepR-biotin (#AF497, R&D): Application:WB,IF,IHC,F; Species Reactivity: Mouse<br>anti-H3 (#4499, CST): Application:WB,IHC,IF,F; Species Reactivity: Human, Mouse, Rat, Monkey

Eukaryotic cell lines

Policy information about cell lines

Cell line source(s)	Cells used in our study are primary MSCs isolated from mice tibiae and femurs.
Authentication	Primary cells were isolated according to well-established protocols and no further authentication was applied.
Mycoplasma contamination	The cell lines were not tested for mycoplasma contamination, but we add Plasmocin prophylactic to prevent mycoplasma contamination.
Commonly misidentified lines (See ICLAC register)	No misidentified cell line was used.

Animals and other organisms

Policy information about studies involving animals; ARRIVE guidelines recommended for reporting animal research

Laboratory animals	Female and male C57BL/6J mice(2/3/4/6/18-month-old) and female Sprague Dawley rats(3/24-month-old) were used in our study. Temperature (23±2°C) and humidity (55%) were held constant in animal housing.
Wild animals	This study did not involve wild animals.
Field-collected samples	This study did not involve samples collected from the field.
Ethics oversight	Approvals for all the protocols were obtained from the Subcommittee on Research and Animal Care (SRAC) of Sichuan University.

Note that full information on the approval of the study protocol must also be provided in the manuscript.

Flow Cytometry

Plots

Confirm that:

- [x] The axis labels state the marker and fluorochrome used (e.g. CD4-FITC).
- [x] The axis scales are clearly visible. Include numbers along axes only for bottom left plot of group (a 'group' is an analysis of identical markers).
- [x] All plots are contour plots with outliers or pseudocolor plots.
- [x] A numerical value for number of cells or percentage (with statistics) is provided.

Methodology

Sample preparation	The mBMSCs were isolated from bone marrow of 18-mon-old mouse tibiae and femurs, and were treated with different concentrations of aKG and vehicle control for 3 days, respectively. The samples were stained using BD Pharmingen™ FITC Annexin V Apoptosis Detection Kit II.
Instrument	The samples were analyzed using BD LSRII.
Software	BD FACSDiva v8.0.3, Flowjo Version 10.5.3
Cell population abundance	N/A. We did not perform cell sorting for specific cell population.
Gating strategy	To detect the apoptotic cells, the gating strategy determined as manufacturer's suggested. Please refer to: https://www.bdbiosciences.com/documents/BD_Research_Apoptosis_FACSArray_AppNote.pdf

- [x] Tick this box to confirm that a figure exemplifying the gating strategy is provided in the Supplementary Information.
